# Structural investigation of *N*-[2-(4-fluoro-3-phen­oxy­benzo­yl)hydrazinecarbo­thio­yl]benzamide and *N*-[2-(4-fluoro-3-phen­oxy­benzo­yl)hydrazinecarbo­thio­yl]-4-meth­oxy­benzamide

**DOI:** 10.1107/S2056989021001900

**Published:** 2021-02-19

**Authors:** Dhananjay Dey, I. Shruti, Deepak Chopra, T. P. Mohan

**Affiliations:** a Department of Chemistry, Indian Institute of Science Education and Research, Bhopal, Bhauri, Bhopal 462066, India; b Rallis India Ltd, Bangalore 560091, Karnataka, India

**Keywords:** crystal structure, drug, hydrogen bonds, mol­ecular conformation, chalcogen-centered inter­actions

## Abstract

The compound *N*-[2-(4-fluoro-3-phen­oxy­benzo­yl)hydrazinecarbo­thio­yl]benzamide, C_21_H_16_FN_3_O_3_S, crystallizes in the monoclinic centrosymmetric space group *P*2_1_/*c* and its mol­ecular conformation is stabilized *via* an intra­molecular N—H⋯O hydrogen bond. The corresponding *para*-meth­oxy derivative, namely, *N*-[2-(4-fluoro-3-phen­oxy­benzo­yl)hydrazinecarbo­thio­yl]-4-meth­oxy­benzamide, C_22_H_18_FN_3_O_4_S, crystallizes in the monoclinic centrosymmetric space group *C*2/*c*. The supra­molecular network mainly comprises N—H⋯O, N—H⋯S and C—H⋯O hydrogen bonds, which contribute towards the formation of the crystal structures for the two mol­ecules. The different inter­molecular inter­actions have been further analysed using Hirshfeld surface analysis and fingerprint plots.

## Abstract 2

Crystal structure analysis of *N*-[2-(4-fluoro-3-phen­oxy­benzo­yl)hydrazinecarbo­thio­yl]benzamide and its 4-meth­oxy derivative highlights the significance of strong and weak hydrogen bonds. The difference in the contributions of atom–atom contacts obtained from Hirshfeld surface analysis and fingerprint plots helps in distinguishing the variations in the crystal packing of the two compounds.

## Chemical context   

Substituted thio­semicarbazides (TSCs) constitute an important class of organic compounds with the general formula *R*–(C=O)–NH–NH–(C=S)–*R*′ and find application in the synthesis of five- and six-membered heterocyclic compounds (Gazieva & Kravchenko, 2012 ▸) and transition-metal complexes (Campbell, 1975 ▸). The chemical diversity of thio­semicarbazides, and their synthesis, including their role in biological applications, is nicely summarized in a recent review article (Acharya *et al.*, 2021 ▸). Di­benzoyl­ated TSCs have been synthesized and explored for their anti­bacterial activity (Qandil *et al.*, 2006 ▸). Furthermore, mol­ecular modelling studies establish the relevance of both geometry and electron-density distribution in the observed anti­bacterial activity (Paneth *et al.*, 2016 ▸). Piperidin-4-yl-based TSCs have been examined for cytotoxicity in breast cancer cell lines in addition to being possible potential topoisomerase inhibitors (Siwek *et al.*, 2014 ▸). 1-(2-Hy­droxy­benzo­yl)-thio­semicarbazides have been observed to exhibit anti­microbial activity and structure–activity relationship (SARs) studies establish that the 2-hy­droxy­benzoyl group plays an important role in enzyme inhibition, in addition to these exhibiting low cytotoxicity (Ameryckx *et al.*, 2018 ▸). Furthermore, triazole-substituted benzoyl­thio­semicarbazides have been synthesized and their effect on the inhibition of corrosion on mild steel has been investigated (Yan *et al.*, 2018 ▸). Keeping in mind the above-mentioned applications of substituted TSCs, we have performed the synthesis and crystal structure analysis of two compounds, namely *N*-[2-(4-fluoro-3-phen­oxy­benzo­yl)hydrazinecarbo­thio­yl]benzamide (A1) and *N*-[2-(4-fluoro-3-phen­oxy­benzo­yl)hydrazinecarbo­thio­yl]-4-meth­oxy­benzamide (A2) in the current study. The mol­ecular conformations have been studied with respect to the various flexible bonds and the occurrence of various inter­molecular inter­actions that contribute towards the stability of the mol­ecules in the crystalline lattice has been investigated in detail *via* an investigation of the crystal packing and qu­anti­tative insights from Hirshfeld surface analysis.
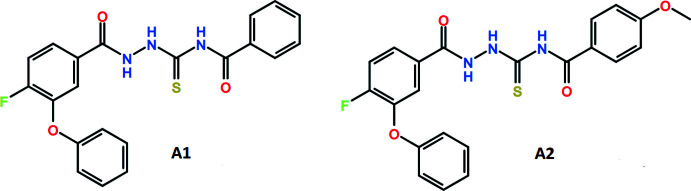


## Structural commentary   

**Figure 1:**
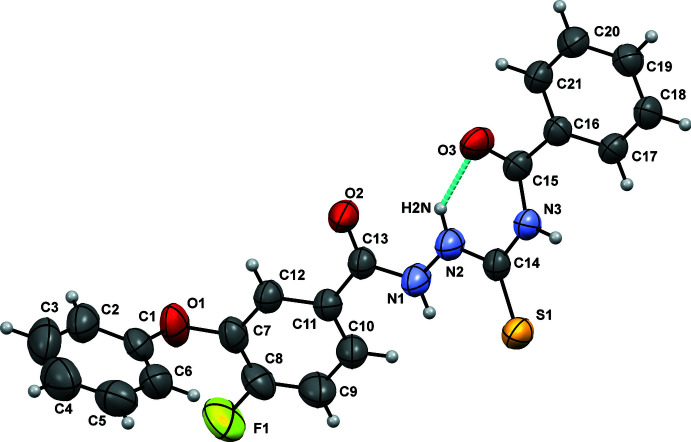
Ellipsoid plot of A1 drawn with 50% ellipsoidal probability. The cyan line indicates the intra­molecular N—H⋯O hydrogen bond.

**Figure 2:**
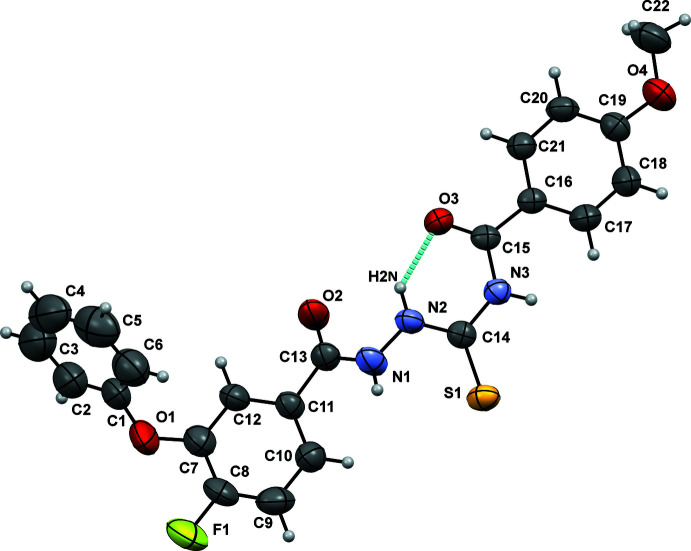
Ellipsoid plot of A2 drawn with 50% ellipsoidal probability. The cyan line indicates the intra­molecular N—H⋯O hydrogen bond.

Compound A1 crystallizes in the centrosymmetric monoclinic *P*2_1_/*c* space group and A2 crystallizes in the centrosymmetric monoclinic *C*2/*c* space group. The mol­ecular structure comprises one fluoro-substituted phen­oxy­benzoyl ring, a rigid and planar (C=O)—NH—NH—(C=S) moiety and a benzamide ring. The bond lengths and bond angles are in accordance with the magnitudes in the literature. The mol­ecular conformations of A1 (Fig. 1 ▸) and A2 (Fig. 2 ▸) are both conformationally locked *via* the presence of an N—H⋯O hydrogen bond (involving H2*N* and O3), the N2⋯O3 distance being 2.555 (2) and 2.589 (4) Å in A1 and A2, respectively. The mol­ecular structure possesses four conformational degrees of freedom due to the free rotation with respect to the N1—N2, C7—O1, O1—C1 and C15—C16 single bonds. The torsion angles C13—N1—N2—C14, C8—C7—O1—C1, C7—O1—C1—C2 and N3—C15—C16—C21 are 163.27 (16)/-143.5 (4)°, 97.3 (2)/149.6 (5)°, 167.18 (18)/148.1 (4)° and −160.26 (15)/-174.7 (3)° in A1/A2, respectively.

## Supra­molecular features   

**Figure 3:**
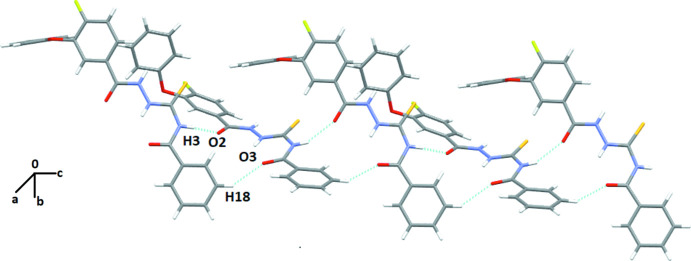
Crystal packing of A1 showing the formation of the crystal structure primarily *via* N—H⋯O and C—H⋯O inter­molecular inter­actions.

**Figure 4:**
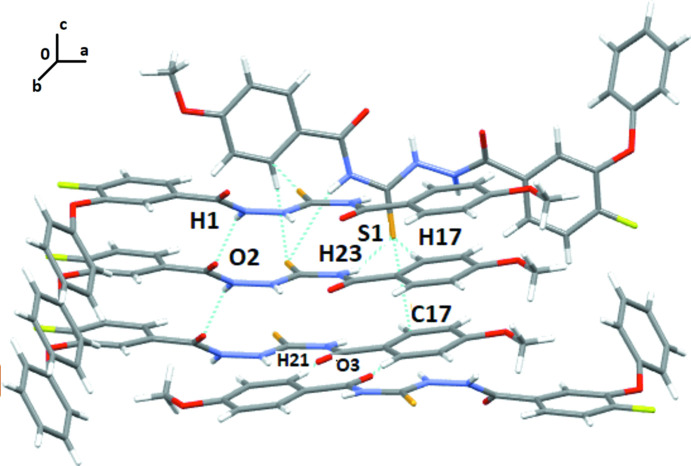
Crystal packing of A2 showing the formation of the crystal structure primarily *via* N—H⋯O, N—H⋯S, C—H⋯S and S⋯C inter­molecular inter­actions.

In the crystal structure of A1, the mol­ecules are primarily assembled through the presence of N3—H3*N*⋯O2 and C18—H18⋯O3 hydrogen bonds (Table 1 ▸), forming mol­ecular chains along the *c*-axis direction utilizing the *c*-glide as the symmetry element (Fig. 3 ▸). Adjacent layers are held together *via* C20—H20⋯O1 and C19—H19⋯S1 hydrogen bonds. The crystal packing of A2 (Fig. 4 ▸) primarily consists of N1—H1⋯O2 hydrogen bonds (Table 2 ▸), forming mol­ecular chains along the *b*-axis direction. Two such adjacent layers are held *via* N3—H3*N*⋯S1 and C17—H17⋯S1 hydrogen bonds. In addition S1⋯C17 contacts (S⋯π type), [3.384 (4) Å, 174.9 (1)°, −*x* + 1, *y* + 1, −*z* + 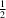] chalcogen-centered contacts are also present in the crystal packing (Fig. 4 ▸). Inter­molecular contacts involving chalcogens are well-recognized in the literature [Pramanik & Chopra, 2020 ▸]. Furthermore, additional C21—H21⋯O3 hydrogen bonds form centrosymmetric dimers and provide additional stability to the crystal packing.

## Database survey   

A search for the di­benzoyl­thio­semicarbazide skeleton, Ph–(C=O)–NH–NH–(C=S)–NH–(C=O)–Ph was carried out in the Cambridge Structural Database (CSD version 5.40, updates of Aug 2019; Groom *et al.*, 2016 ▸) . No hits were obtained. Thus, further systematic studies related to the investigation of the role of differently substituted thio­semicarbazide mol­ecules towards the crystal packing, including a detailed investigation of polymorphism in this class of compounds, is of relevance.

## Hirshfeld surface analysis and fingerprint plots   

**Figure 5:**
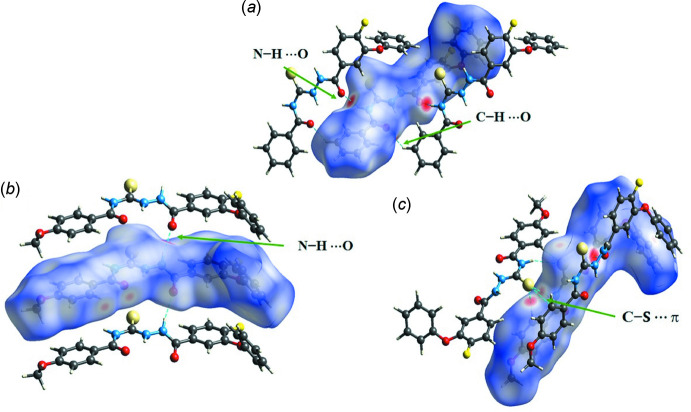
The Hirshfeld surface mapped over *d*_norm_ for (*a*) A1, (*b*) A2 depicting N—H⋯O hydrogen bonds and (*c*) A2 depicting C—S⋯π inter­actions.

**Figure 6:**
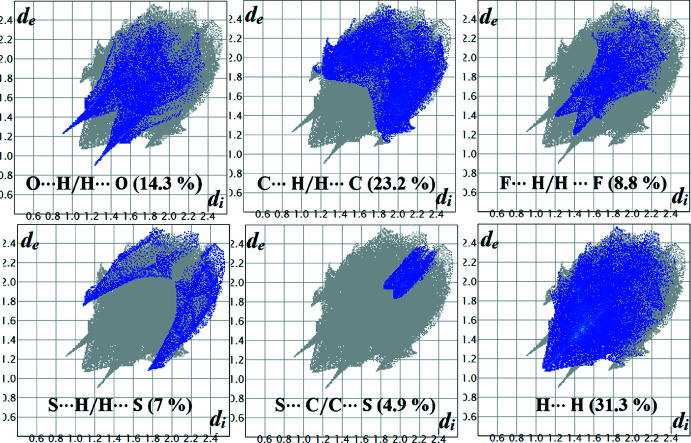
The fingerprint plots for A1 showing the different contributions derived from the H⋯H, C⋯H/H⋯C, O⋯H/H⋯O, H⋯F/F⋯H,S⋯H/S⋯H and C⋯S/S⋯C contacts.

**Figure 7:**
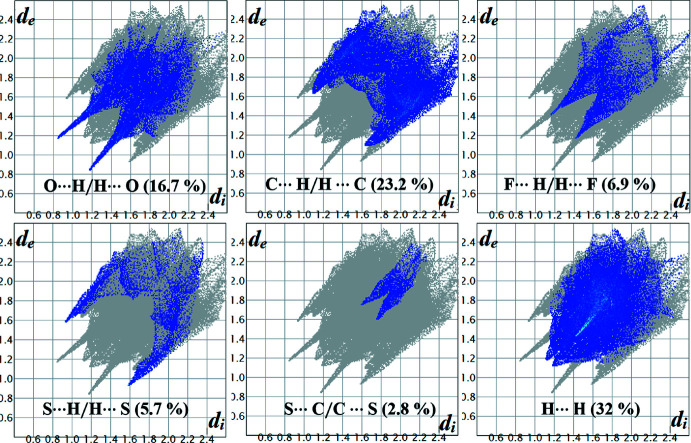
The fingerprint plots for A2 showing the different contributions derived from the H⋯H, C⋯H/H⋯C, O⋯H/H⋯O, H⋯F/F⋯H,S⋯H/S⋯H and C⋯S/S⋯C contacts.

The relevance of different inter­molecular inter­actions can be established *via* Hirshfeld surface analysis (Spackman & Jayatilaka, 2009 ▸). These surfaces, along with the two-dimensional fingerprint plots, were evaluated using *Crystal Explorer 17.5* (Turner *et al.*, 2017 ▸). The surfaces mapped over *d*_norm_ for A1, Fig. 5 ▸(*a*), and A2, Fig. 5 ▸(*b*) and 5(*c*), show the important hydrogen bonds. The red and blue spots correspond to inter­molecular inter­actions that are less or greater than the sum of the van der Waals radii. The fingerprint plots depict the individual contributions of the different inter­actions. The fingerprint plots for A1/A2 (Figs. 6 ▸ and 7 ▸) show that the greatest contributions are from H⋯H (31.3/32%) contacts, followed by C⋯H/H⋯C (23.2/23.2%), O⋯H/H⋯O (14.3/16.7%), S⋯H/H⋯S (7/5.7%), S⋯C/C⋯S (4.9/2.8%) and F⋯H/H⋯F (8.8/6.9%) contacts. The O⋯H/H⋯O contribution is slightly higher in the case of A2 (16.7%) due to the presence of an additional meth­oxy group in the mol­ecule. Further inter­actions, involving F⋯H/H⋯F, contributing around 7–9% (A1: 8.8% and A2: 6.9%) and S⋯H (A1: 7.0% and A2: 5.7%) correspond to the presence of highly directional inter­actions, involving fluorine and sulfur in A2, and are important; this is clearly illustrated in the fingerprint plot (Fig. 7 ▸). The percentage contribution of S⋯C/C⋯S contacts in A2 is 2.8% lower than in A1. However, the relevance of this contact is greater in A2 on account of the presence of the highly directional C—S⋯π inter­molecular contact and this feature is also clearly visible in the 2D fingerprint plot (Fig. 7 ▸).

## Synthesis and Crystallization   

The title compounds were synthesized in accordance with the procedure reported in the literature (Mohan, 2006 ▸). Crystallization was performed in 5.0 ml beakers at room temperature *via* the slow evaporation method from methanol solvent.

## Refinement   

Crystal data, data collection and structure refinement details are summarized in Table 3 ▸. H atoms were placed in idealized positions (N—H = 0.86 Å, C—H = 0.93 Å) and refined using a riding model with *U*_iso_(H) = 1.2*U*_eq_(C, N) or 1.5*U*_eq_(C-meth­yl).

## Supplementary Material

Crystal structure: contains datablock(s) A1, A2. DOI: 10.1107/S2056989021001900/dj2021sup1.cif

Structure factors: contains datablock(s) A1. DOI: 10.1107/S2056989021001900/dj2021A1sup2.hkl

Structure factors: contains datablock(s) A2. DOI: 10.1107/S2056989021001900/dj2021A2sup3.hkl

Supporting information file. DOI: 10.1107/S2056989021001900/dj2021A1sup4.cml

Supporting information file. DOI: 10.1107/S2056989021001900/dj2021A2sup5.cml

CCDC references: 2063226, 2063225

Additional supporting information:  crystallographic information; 3D view; checkCIF report

## Figures and Tables

**Table 1:** Hydrogen-bond geometry (Å, °) for A1

*D*—H⋯*A*	*D*—H	H⋯*A*	*D*⋯*A*	*D*—H⋯*A*
N2—H2*N*⋯O3	0.86	1.88	2.555 (2)	135
C18—H18⋯O3^i^	0.93	2.45	3.218 (2)	141
N3—H3*N*⋯O2^i^	0.86	2.28	3.067 (2)	152
C19—H19⋯S1^ii^	0.93	2.98	3.778 (2)	145
C20—H20⋯O1^iii^	0.93	2.77	3.510 (3)	138

Symmetry codes: (i) x, -y+{\script{1\over 2}}, z+{\script{1\over 2}}; (ii) -x+1, y+{\script{1\over 2}}, -z+{\script{5\over 2}}; (iii) -x+1, y+{\script{1\over 2}}, -z+{\script{3\over 2}}.

**Table 2:** Hydrogen-bond geometry (Å, °) for A2

*D*—H⋯*A*	*D*—H	H⋯*A*	*D*⋯*A*	*D*—H⋯*A*
N2—H2*N*⋯O3	0.86	1.92	2.589 (4)	134
N3—H3*N*⋯S1^i^	0.86	2.80	3.615 (3)	159
C17—H17⋯S1^i^	0.93	2.69	3.614 (4)	174
N1—H1⋯O2^ii^	0.86	2.15	2.915 (4)	148
C21—H21⋯O3^iii^	0.93	2.57	3.399 (4)	148

Symmetry codes: (i) -x+1, y, -z+{\script{1\over 2}}; (ii) x, y+1, z; (iii) -x+1, -y-1, -z.

**Table 3:** Experimental details

	A1	A2
Crystal data		
Chemical formula	C_21_H_16_FN_3_O_3_S	C_22_H_18_FN_3_O_4_S
*M*_r_	409.43	439.45
Crystal system, space group	Monoclinic, *P*2_1_/*c*	Monoclinic, *C*2/*c*
Temperature (K)	298	298
*a*, *b*, *c* (Å)	18.3849 (13), 7.7063 (6), 13.9216 (10)	47.298 (3), 4.8054 (3), 18.4939 (10)
β (°)	100.136 (5)	100.429 (6)
*V* (Å^3^)	1941.6 (2)	4134.0 (4)
*Z*	4	8
Radiation type	Mo *K*α	Mo *K*α
μ (mm^−1^)	0.20	0.20
Crystal size (mm)	0.27 × 0.20 × 0.14	0.25 × 0.17 × 0.10

Data collection		
Diffractometer	Bruker APEXII CCD	Bruker APEXII CCD
Absorption correction	Multi-scan (*SADABS*; Bruker, 2008 ▸)	Multi-scan (*SADABS*; Bruker, 2008 ▸)
No. of measured, independent and observed [*I* > 2σ(*I*)] reflections	31160, 4460, 2753	9841, 2188, 1751
*R*_int_	0.043	0.062
θ_max_ (°)	27.7	20.9
(sin θ/λ)_max_ (Å^−1^)	0.653	0.503

Refinement		
*R*[*F* ^2^ > 2σ(*F* ^2^)], *wR*(*F* ^2^), *S*	0.043, 0.119, 1.04	0.046, 0.122, 1.06
No. of reflections	4460	2188
No. of parameters	262	281
H-atom treatment	H-atom parameters constrained	H-atom parameters constrained
Δρ_max_, Δρ_min_ (e Å^−3^)	0.15, −0.17	0.20, −0.16

Computer programs: *APEX2* (Bruker, 2012 ▸), *SAINT* (Bruker, 2008 ▸), *SHELXS97* (Sheldrick, 2008 ▸), *SHELXL2018/3* (Sheldrick, 2015 ▸), *Mercury* (Macrae *et al.*, 2020 ▸), *WinGX* (Farrugia, 2012 ▸) and *PLATON* (Spek, 2020 ▸).

## supplementary crystallographic information

### N-[2-(4-Fluoro-3-phenoxybenzoyl)hydrazinecarbothioyl]benzamide (A1) Crystal data

**Table:**

C_21_H_16_FN_3_O_3_S	*F*(000) = 848
*M**_r_* = 409.43	*D*_x_ = 1.401 Mg m^−^^3^
Monoclinic, *P*2_1_/*c*	Mo *K*α radiation, λ = 0.71073 Å
*a* = 18.3849 (13) Å	Cell parameters from 10485 reflections
*b* = 7.7063 (6) Å	θ = 2.2–28.6°
*c* = 13.9216 (10) Å	µ = 0.20 mm^−^^1^
β = 100.136 (5)°	*T* = 298 K
*V* = 1941.6 (2) Å^3^	Plates, colorless
*Z* = 4	0.27 × 0.20 × 0.14 mm

### N-[2-(4-Fluoro-3-phenoxybenzoyl)hydrazinecarbothioyl]benzamide (A1) Data collection

**Table:**

Bruker APEXII CCD diffractometer	2753 reflections with *I* > 2σ(*I*)
φ and ω scans	*R*_int_ = 0.043
Absorption correction: multi-scan (SADABS; Bruker, 2008)	θ_max_ = 27.7°, θ_min_ = 2.3°
	*h* = −23→21
31160 measured reflections	*k* = −10→10
4460 independent reflections	*l* = −18→18

### N-[2-(4-Fluoro-3-phenoxybenzoyl)hydrazinecarbothioyl]benzamide (A1) Refinement

**Table:**

Refinement on *F*^2^	Primary atom site location: structure-invariant direct methods
Least-squares matrix: full	Hydrogen site location: inferred from neighbouring sites
*R*[*F*^2^ > 2σ(*F*^2^)] = 0.043	H-atom parameters constrained
*wR*(*F*^2^) = 0.119	*w* = 1/[σ^2^(*F*_o_^2^) + (0.0531*P*)^2^ + 0.1773*P*] where *P* = (*F*_o_^2^ + 2*F*_c_^2^)/3
*S* = 1.04	(Δ/σ)_max_ < 0.001
4460 reflections	Δρ_max_ = 0.15 e Å^−^^3^
262 parameters	Δρ_min_ = −0.17 e Å^−^^3^
0 restraints	

### N-[2-(4-Fluoro-3-phenoxybenzoyl)hydrazinecarbothioyl]benzamide (A1) Special details

**Table:**

Geometry. All esds (except the esd in the dihedral angle between two l.s. planes) are estimated using the full covariance matrix. The cell esds are taken into account individually in the estimation of esds in distances, angles and torsion angles; correlations between esds in cell parameters are only used when they are defined by crystal symmetry. An approximate (isotropic) treatment of cell esds is used for estimating esds involving l.s. planes.

### N-[2-(4-Fluoro-3-phenoxybenzoyl)hydrazinecarbothioyl]benzamide (A1) Fractional atomic coordinates and isotropic or equivalent isotropic displacement parameters (Å^2^)

**Table:**

	*x*	*y*	*z*	*U*_iso_*/*U*_eq_	
N2	0.35731 (8)	0.0786 (2)	0.88306 (10)	0.0603 (4)	
H2N	0.398146	0.108884	0.865793	0.072*	
O2	0.35727 (7)	0.08997 (19)	0.69344 (9)	0.0715 (4)	
N1	0.30299 (8)	0.0015 (2)	0.81656 (10)	0.0635 (4)	
H1	0.267495	−0.053998	0.835584	0.076*	
C15	0.47522 (9)	0.2198 (2)	1.01326 (12)	0.0544 (4)	
N3	0.40609 (7)	0.18548 (18)	1.03435 (9)	0.0538 (4)	
H3N	0.398570	0.216229	1.091172	0.065*	
C19	0.63953 (10)	0.4486 (3)	1.22873 (13)	0.0637 (5)	
H19	0.675815	0.500499	1.274731	0.076*	
C13	0.30627 (10)	0.0143 (2)	0.72070 (12)	0.0546 (4)	
C11	0.24397 (10)	−0.0680 (2)	0.65341 (12)	0.0548 (4)	
O3	0.48982 (7)	0.18773 (19)	0.93243 (8)	0.0737 (4)	
C12	0.23455 (10)	−0.0202 (2)	0.55602 (12)	0.0587 (5)	
H12	0.267655	0.056422	0.535242	0.070*	
C18	0.58177 (10)	0.3634 (3)	1.25799 (13)	0.0651 (5)	
H18	0.579476	0.355776	1.324057	0.078*	
C16	0.53052 (9)	0.2979 (2)	1.09115 (11)	0.0509 (4)	
C7	0.17640 (11)	−0.0857 (3)	0.48974 (13)	0.0650 (5)	
C17	0.52695 (10)	0.2888 (2)	1.19000 (12)	0.0589 (5)	
H17	0.487572	0.232436	1.210329	0.071*	
O1	0.16903 (8)	−0.0470 (2)	0.39147 (9)	0.0815 (4)	
C20	0.64377 (11)	0.4571 (3)	1.13081 (13)	0.0683 (5)	
H20	0.683089	0.514503	1.110943	0.082*	
C14	0.34729 (9)	0.1066 (2)	0.97367 (11)	0.0524 (4)	
C1	0.13186 (10)	0.1053 (3)	0.35731 (13)	0.0658 (5)	
C10	0.19555 (10)	−0.1858 (3)	0.68345 (14)	0.0678 (5)	
H10	0.202440	−0.221053	0.748315	0.081*	
C21	0.59024 (10)	0.3814 (3)	1.06281 (12)	0.0610 (5)	
H21	0.593940	0.385998	0.997084	0.073*	
C8	0.12811 (11)	−0.1982 (3)	0.52212 (15)	0.0755 (6)	
F1	0.07018 (8)	−0.2577 (2)	0.45739 (10)	0.1157 (5)	
C9	0.13740 (12)	−0.2507 (3)	0.61757 (16)	0.0828 (6)	
H9	0.104704	−0.329362	0.637526	0.099*	
C6	0.09068 (11)	0.2008 (3)	0.41052 (15)	0.0756 (6)	
H6	0.086151	0.166959	0.473327	0.091*	
C4	0.06114 (16)	0.3971 (4)	0.2770 (2)	0.1119 (9)	
H4	0.036467	0.495305	0.249363	0.134*	
C2	0.13978 (13)	0.1537 (3)	0.26449 (15)	0.0849 (7)	
H2	0.169036	0.089353	0.229477	0.102*	
C3	0.10316 (18)	0.3001 (4)	0.2249 (2)	0.1101 (9)	
H3	0.107028	0.333605	0.161870	0.132*	
C5	0.05585 (13)	0.3485 (4)	0.3694 (2)	0.0958 (7)	
H5	0.028365	0.415792	0.405426	0.115*	
S1	0.26986 (3)	0.05350 (8)	1.01272 (3)	0.07183 (19)	

### N-[2-(4-Fluoro-3-phenoxybenzoyl)hydrazinecarbothioyl]benzamide (A1) Atomic displacement parameters (Å^2^)

**Table:**

	*U*^11^	*U*^22^	*U*^33^	*U*^12^	*U*^13^	*U*^23^
N2	0.0605 (9)	0.0788 (10)	0.0420 (8)	−0.0123 (8)	0.0097 (7)	−0.0069 (7)
O2	0.0662 (8)	0.0981 (10)	0.0499 (7)	−0.0220 (7)	0.0097 (6)	0.0030 (7)
N1	0.0659 (10)	0.0808 (11)	0.0435 (8)	−0.0197 (8)	0.0090 (7)	−0.0051 (8)
C15	0.0558 (10)	0.0637 (11)	0.0457 (9)	0.0021 (8)	0.0147 (8)	−0.0033 (8)
N3	0.0539 (8)	0.0702 (10)	0.0381 (7)	−0.0016 (7)	0.0100 (6)	−0.0055 (7)
C19	0.0562 (11)	0.0809 (13)	0.0525 (10)	−0.0022 (10)	0.0053 (8)	−0.0055 (10)
C13	0.0590 (11)	0.0618 (11)	0.0425 (9)	−0.0026 (9)	0.0076 (8)	0.0010 (8)
C11	0.0566 (10)	0.0612 (11)	0.0457 (9)	−0.0014 (9)	0.0070 (8)	−0.0016 (8)
O3	0.0684 (8)	0.1078 (11)	0.0495 (7)	−0.0126 (7)	0.0229 (6)	−0.0201 (7)
C12	0.0637 (11)	0.0683 (12)	0.0444 (9)	−0.0035 (9)	0.0107 (8)	−0.0032 (8)
C18	0.0610 (11)	0.0937 (14)	0.0408 (9)	0.0028 (10)	0.0095 (8)	−0.0040 (9)
C16	0.0512 (10)	0.0590 (10)	0.0433 (8)	0.0056 (8)	0.0104 (7)	−0.0012 (8)
C7	0.0693 (12)	0.0775 (13)	0.0450 (10)	0.0071 (10)	0.0012 (9)	−0.0073 (9)
C17	0.0540 (10)	0.0781 (12)	0.0467 (9)	−0.0011 (9)	0.0149 (8)	−0.0006 (9)
O1	0.0998 (11)	0.0991 (11)	0.0421 (7)	0.0170 (9)	0.0026 (7)	−0.0105 (7)
C20	0.0595 (11)	0.0891 (14)	0.0576 (11)	−0.0121 (10)	0.0136 (9)	0.0041 (10)
C14	0.0585 (11)	0.0573 (10)	0.0409 (8)	0.0019 (8)	0.0076 (8)	0.0015 (8)
C1	0.0568 (11)	0.0850 (14)	0.0496 (10)	−0.0092 (10)	−0.0068 (9)	−0.0020 (10)
C10	0.0663 (12)	0.0775 (13)	0.0573 (11)	−0.0110 (10)	0.0048 (9)	0.0084 (10)
C21	0.0609 (11)	0.0792 (13)	0.0443 (9)	−0.0033 (10)	0.0134 (8)	0.0014 (9)
C8	0.0648 (13)	0.0863 (15)	0.0666 (13)	−0.0101 (11)	−0.0126 (10)	−0.0100 (11)
F1	0.0972 (10)	0.1390 (12)	0.0957 (9)	−0.0334 (9)	−0.0246 (8)	−0.0135 (9)
C9	0.0714 (13)	0.0906 (15)	0.0824 (15)	−0.0234 (12)	0.0023 (11)	0.0080 (13)
C6	0.0658 (13)	0.0965 (17)	0.0625 (12)	0.0016 (11)	0.0058 (10)	−0.0011 (12)
C4	0.101 (2)	0.112 (2)	0.116 (2)	0.0046 (17)	0.0013 (18)	0.034 (2)
C2	0.0881 (16)	0.1073 (18)	0.0569 (12)	−0.0178 (14)	0.0063 (11)	−0.0009 (13)
C3	0.128 (2)	0.122 (2)	0.0745 (16)	−0.0238 (19)	0.0032 (16)	0.0288 (17)
C5	0.0718 (15)	0.1026 (19)	0.111 (2)	0.0045 (14)	0.0110 (14)	0.0038 (16)
S1	0.0630 (3)	0.1025 (4)	0.0515 (3)	−0.0143 (3)	0.0141 (2)	−0.0031 (3)

### N-[2-(4-Fluoro-3-phenoxybenzoyl)hydrazinecarbothioyl]benzamide (A1) Geometric parameters (Å, º)

**Table:**

N2—C14	1.3242 (19)	C7—O1	1.383 (2)
N2—N1	1.3718 (19)	C17—H17	0.9300
N2—H2N	0.8600	O1—C1	1.399 (2)
O2—C13	1.220 (2)	C20—C21	1.370 (3)
N1—C13	1.350 (2)	C20—H20	0.9300
N1—H1	0.8600	C14—S1	1.6617 (17)
C15—O3	1.2272 (18)	C1—C6	1.364 (3)
C15—N3	1.379 (2)	C1—C2	1.377 (3)
C15—C16	1.478 (2)	C10—C9	1.374 (3)
N3—C14	1.389 (2)	C10—H10	0.9300
N3—H3N	0.8600	C21—H21	0.9300
C19—C18	1.370 (3)	C8—F1	1.349 (2)
C19—C20	1.381 (2)	C8—C9	1.371 (3)
C19—H19	0.9300	C9—H9	0.9300
C13—C11	1.488 (2)	C6—C5	1.380 (3)
C11—C12	1.386 (2)	C6—H6	0.9300
C11—C10	1.387 (2)	C4—C5	1.360 (4)
C12—C7	1.379 (3)	C4—C3	1.370 (4)
C12—H12	0.9300	C4—H4	0.9300
C18—C17	1.381 (2)	C2—C3	1.378 (4)
C18—H18	0.9300	C2—H2	0.9300
C16—C21	1.389 (2)	C3—H3	0.9300
C16—C17	1.391 (2)	C5—H5	0.9300
C7—C8	1.372 (3)		
			
C14—N2—N1	120.44 (14)	C21—C20—C19	120.23 (17)
C14—N2—H2N	119.8	C21—C20—H20	119.9
N1—N2—H2N	119.8	C19—C20—H20	119.9
C13—N1—N2	118.79 (14)	N2—C14—N3	115.25 (14)
C13—N1—H1	120.6	N2—C14—S1	122.85 (13)
N2—N1—H1	120.6	N3—C14—S1	121.90 (12)
O3—C15—N3	120.99 (16)	C6—C1—C2	121.6 (2)
O3—C15—C16	121.44 (15)	C6—C1—O1	123.55 (18)
N3—C15—C16	117.57 (13)	C2—C1—O1	114.8 (2)
C15—N3—C14	127.02 (13)	C9—C10—C11	120.14 (18)
C15—N3—H3N	116.5	C9—C10—H10	119.9
C14—N3—H3N	116.5	C11—C10—H10	119.9
C18—C19—C20	119.86 (17)	C20—C21—C16	120.58 (16)
C18—C19—H19	120.1	C20—C21—H21	119.7
C20—C19—H19	120.1	C16—C21—H21	119.7
O2—C13—N1	120.86 (16)	F1—C8—C9	119.7 (2)
O2—C13—C11	123.81 (15)	F1—C8—C7	118.42 (19)
N1—C13—C11	115.33 (15)	C9—C8—C7	121.84 (18)
C12—C11—C10	119.51 (17)	C8—C9—C10	119.28 (19)
C12—C11—C13	116.91 (16)	C8—C9—H9	120.4
C10—C11—C13	123.57 (16)	C10—C9—H9	120.4
C7—C12—C11	120.40 (18)	C1—C6—C5	118.7 (2)
C7—C12—H12	119.8	C1—C6—H6	120.6
C11—C12—H12	119.8	C5—C6—H6	120.6
C19—C18—C17	120.41 (16)	C5—C4—C3	119.4 (3)
C19—C18—H18	119.8	C5—C4—H4	120.3
C17—C18—H18	119.8	C3—C4—H4	120.3
C21—C16—C17	118.81 (16)	C1—C2—C3	118.1 (2)
C21—C16—C15	117.16 (14)	C1—C2—H2	120.9
C17—C16—C15	124.01 (15)	C3—C2—H2	120.9
C8—C7—C12	118.78 (17)	C4—C3—C2	121.1 (2)
C8—C7—O1	120.31 (18)	C4—C3—H3	119.4
C12—C7—O1	120.81 (18)	C2—C3—H3	119.4
C18—C17—C16	120.09 (16)	C4—C5—C6	121.0 (3)
C18—C17—H17	120.0	C4—C5—H5	119.5
C16—C17—H17	120.0	C6—C5—H5	119.5
C7—O1—C1	118.30 (15)		
			
C14—N2—N1—C13	163.27 (16)	N1—N2—C14—S1	−0.5 (2)
O3—C15—N3—C14	3.4 (3)	C15—N3—C14—N2	−7.4 (2)
C16—C15—N3—C14	−177.17 (15)	C15—N3—C14—S1	173.17 (14)
N2—N1—C13—O2	1.4 (3)	C7—O1—C1—C6	−12.8 (3)
N2—N1—C13—C11	−178.11 (15)	C7—O1—C1—C2	167.18 (18)
O2—C13—C11—C12	−15.9 (3)	C12—C11—C10—C9	−1.9 (3)
N1—C13—C11—C12	163.59 (16)	C13—C11—C10—C9	177.23 (19)
O2—C13—C11—C10	164.89 (19)	C19—C20—C21—C16	1.1 (3)
N1—C13—C11—C10	−15.6 (3)	C17—C16—C21—C20	−1.5 (3)
C10—C11—C12—C7	1.7 (3)	C15—C16—C21—C20	179.92 (17)
C13—C11—C12—C7	−177.53 (17)	C12—C7—C8—F1	178.13 (18)
C20—C19—C18—C17	−1.2 (3)	O1—C7—C8—F1	−5.5 (3)
O3—C15—C16—C21	19.2 (3)	C12—C7—C8—C9	−1.9 (3)
N3—C15—C16—C21	−160.26 (15)	O1—C7—C8—C9	174.4 (2)
O3—C15—C16—C17	−159.26 (18)	F1—C8—C9—C10	−178.4 (2)
N3—C15—C16—C17	21.3 (3)	C7—C8—C9—C10	1.7 (4)
C11—C12—C7—C8	0.2 (3)	C11—C10—C9—C8	0.3 (3)
C11—C12—C7—O1	−176.13 (16)	C2—C1—C6—C5	0.5 (3)
C19—C18—C17—C16	0.8 (3)	O1—C1—C6—C5	−179.55 (18)
C21—C16—C17—C18	0.6 (3)	C6—C1—C2—C3	−1.7 (3)
C15—C16—C17—C18	179.02 (17)	O1—C1—C2—C3	178.36 (19)
C8—C7—O1—C1	97.3 (2)	C5—C4—C3—C2	0.4 (4)
C12—C7—O1—C1	−86.4 (2)	C1—C2—C3—C4	1.3 (4)
C18—C19—C20—C21	0.3 (3)	C3—C4—C5—C6	−1.6 (4)
N1—N2—C14—N3	−179.95 (15)	C1—C6—C5—C4	1.2 (4)

### N-[2-(4-Fluoro-3-phenoxybenzoyl)hydrazinecarbothioyl]benzamide (A1) Hydrogen-bond geometry (Å, º)

**Table:**

*D*—H···*A*	*D*—H	H···*A*	*D*···*A*	*D*—H···*A*
N2—H2*N*···O3	0.86	1.88	2.555 (2)	135
C18—H18···O3^i^	0.93	2.45	3.218 (2)	141
N3—H3*N*···O2^i^	0.86	2.28	3.067 (2)	152
C19—H19···S1^ii^	0.93	2.98	3.778 (2)	145
C20—H20···O1^iii^	0.93	2.77	3.510 (3)	138

Symmetry codes: (i) *x*, −*y*+1/2, *z*+1/2; (ii) −*x*+1, *y*+1/2, −*z*+5/2; (iii) −*x*+1, *y*+1/2, −*z*+3/2.

### N-[2-(4-Fluoro-3-phenoxybenzoyl)hydrazinecarbothioyl]-4-methoxybenzamide (A2) Crystal data

**Table:**

C_22_H_18_FN_3_O_4_S	*F*(000) = 1824
*M**_r_* = 439.45	*D*_x_ = 1.412 Mg m^−^^3^
Monoclinic, *C*2/*c*	Mo *K*α radiation, λ = 0.71073 Å
*a* = 47.298 (3) Å	Cell parameters from 9864 reflections
*b* = 4.8054 (3) Å	θ = 2.3–21.0°
*c* = 18.4939 (10) Å	µ = 0.20 mm^−^^1^
β = 100.429 (6)°	*T* = 298 K
*V* = 4134.0 (4) Å^3^	Plates, colorless
*Z* = 8	0.25 × 0.17 × 0.10 mm

### N-[2-(4-Fluoro-3-phenoxybenzoyl)hydrazinecarbothioyl]-4-methoxybenzamide (A2) Data collection

**Table:**

Bruker APEXII CCD diffractometer	1751 reflections with *I* > 2σ(*I*)
φ and ω scans	*R*_int_ = 0.062
Absorption correction: multi-scan (SADABS; Bruker, 2008)	θ_max_ = 20.9°, θ_min_ = 2.3°
	*h* = −46→38
9841 measured reflections	*k* = −4→4
2188 independent reflections	*l* = −18→18

### N-[2-(4-Fluoro-3-phenoxybenzoyl)hydrazinecarbothioyl]-4-methoxybenzamide (A2) Refinement

**Table:**

Refinement on *F*^2^	Primary atom site location: structure-invariant direct methods
Least-squares matrix: full	Hydrogen site location: inferred from neighbouring sites
*R*[*F*^2^ > 2σ(*F*^2^)] = 0.046	H-atom parameters constrained
*wR*(*F*^2^) = 0.122	*w* = 1/[σ^2^(*F*_o_^2^) + (0.0421*P*)^2^ + 7.4575*P*] where *P* = (*F*_o_^2^ + 2*F*_c_^2^)/3
*S* = 1.06	(Δ/σ)_max_ < 0.001
2188 reflections	Δρ_max_ = 0.20 e Å^−^^3^
281 parameters	Δρ_min_ = −0.16 e Å^−^^3^
0 restraints	

### N-[2-(4-Fluoro-3-phenoxybenzoyl)hydrazinecarbothioyl]-4-methoxybenzamide (A2) Special details

**Table:**

Geometry. All esds (except the esd in the dihedral angle between two l.s. planes) are estimated using the full covariance matrix. The cell esds are taken into account individually in the estimation of esds in distances, angles and torsion angles; correlations between esds in cell parameters are only used when they are defined by crystal symmetry. An approximate (isotropic) treatment of cell esds is used for estimating esds involving l.s. planes.

### N-[2-(4-Fluoro-3-phenoxybenzoyl)hydrazinecarbothioyl]-4-methoxybenzamide (A2) Fractional atomic coordinates and isotropic or equivalent isotropic displacement parameters (Å^2^)

**Table:**

	*x*	*y*	*z*	*U*_iso_*/*U*_eq_	
S1	0.46155 (2)	0.5067 (2)	0.20257 (5)	0.0570 (4)	
O3	0.48485 (5)	−0.1767 (5)	0.05581 (13)	0.0564 (7)	
O2	0.40193 (5)	−0.2064 (6)	0.06269 (16)	0.0693 (8)	
N3	0.49466 (6)	0.1596 (6)	0.14319 (15)	0.0476 (8)	
H3N	0.508458	0.244009	0.171425	0.057*	
O4	0.61871 (6)	−0.3864 (7)	0.15455 (17)	0.0846 (9)	
N2	0.44651 (6)	0.1428 (6)	0.09635 (17)	0.0548 (8)	
H2N	0.450510	0.022383	0.065314	0.066*	
F1	0.28801 (5)	0.3872 (7)	0.11032 (16)	0.1091 (10)	
C16	0.53329 (7)	−0.1387 (7)	0.11626 (18)	0.0431 (9)	
N1	0.41856 (7)	0.2174 (7)	0.09631 (18)	0.0636 (9)	
H1	0.414588	0.386840	0.105555	0.076*	
C13	0.39757 (8)	0.0319 (9)	0.0822 (2)	0.0514 (10)	
C11	0.36866 (8)	0.1329 (8)	0.0915 (2)	0.0516 (10)	
C14	0.46729 (8)	0.2578 (7)	0.14456 (18)	0.0438 (9)	
O1	0.29250 (6)	0.0013 (8)	0.01236 (19)	0.1023 (12)	
C21	0.54221 (8)	−0.3385 (8)	0.0720 (2)	0.0547 (10)	
H21	0.528867	−0.416711	0.034349	0.066*	
C15	0.50265 (8)	−0.0588 (7)	0.10183 (19)	0.0441 (9)	
C19	0.59029 (8)	−0.3153 (9)	0.1383 (2)	0.0576 (10)	
C20	0.57034 (9)	−0.4253 (8)	0.0820 (2)	0.0603 (11)	
H20	0.575894	−0.558120	0.050737	0.072*	
C17	0.55367 (9)	−0.0281 (8)	0.1725 (2)	0.0601 (11)	
H17	0.548250	0.107588	0.203190	0.072*	
C12	0.34481 (8)	0.0178 (9)	0.0473 (2)	0.0638 (11)	
H12	0.347192	−0.119851	0.013548	0.077*	
C18	0.58180 (9)	−0.1171 (10)	0.1835 (2)	0.0678 (12)	
H18	0.595147	−0.042543	0.221674	0.081*	
C10	0.36521 (9)	0.3291 (9)	0.1432 (2)	0.0663 (11)	
H10	0.381175	0.405703	0.173417	0.080*	
C7	0.31751 (9)	0.1059 (10)	0.0528 (2)	0.0710 (12)	
C8	0.31463 (9)	0.3024 (10)	0.1041 (3)	0.0714 (12)	
C9	0.33763 (11)	0.4125 (10)	0.1500 (3)	0.0804 (14)	
H9	0.334955	0.542017	0.185500	0.096*	
C1	0.29108 (10)	−0.0900 (12)	−0.0591 (3)	0.0824 (15)	
C22	0.62909 (10)	−0.5808 (11)	0.1078 (3)	0.0994 (17)	
H22A	0.619623	−0.756318	0.110327	0.149*	
H22B	0.649439	−0.604267	0.123394	0.149*	
H22C	0.625228	−0.513264	0.058166	0.149*	
C2	0.27240 (11)	−0.3041 (13)	−0.0798 (3)	0.1014 (17)	
H2	0.262932	−0.386389	−0.045353	0.122*	
C5	0.30114 (16)	−0.0664 (17)	−0.1801 (4)	0.120 (2)	
H5	0.310776	0.012404	−0.214659	0.144*	
C4	0.28168 (18)	−0.2822 (19)	−0.1998 (4)	0.128 (3)	
H4	0.278282	−0.348577	−0.247824	0.153*	
C6	0.30602 (11)	0.0306 (13)	−0.1075 (3)	0.1001 (17)	
H6	0.319071	0.173145	−0.092598	0.120*	
C3	0.26762 (14)	−0.3964 (17)	−0.1492 (5)	0.131 (2)	
H3	0.254553	−0.539896	−0.162868	0.157*	

### N-[2-(4-Fluoro-3-phenoxybenzoyl)hydrazinecarbothioyl]-4-methoxybenzamide (A2) Atomic displacement parameters (Å^2^)

**Table:**

	*U*^11^	*U*^22^	*U*^33^	*U*^12^	*U*^13^	*U*^23^
S1	0.0682 (7)	0.0481 (6)	0.0553 (6)	0.0142 (5)	0.0124 (5)	−0.0014 (5)
O3	0.0554 (16)	0.0545 (17)	0.0589 (16)	0.0012 (14)	0.0092 (13)	−0.0131 (14)
O2	0.0576 (17)	0.0393 (18)	0.109 (2)	0.0029 (14)	0.0092 (15)	−0.0124 (16)
N3	0.0473 (19)	0.0409 (18)	0.0556 (18)	−0.0008 (16)	0.0119 (14)	−0.0034 (16)
O4	0.0587 (19)	0.094 (2)	0.098 (2)	0.0215 (18)	0.0072 (16)	−0.0099 (19)
N2	0.047 (2)	0.0453 (19)	0.074 (2)	0.0059 (17)	0.0161 (17)	−0.0125 (17)
F1	0.0701 (18)	0.141 (3)	0.127 (2)	0.0232 (18)	0.0454 (15)	−0.0117 (19)
C16	0.051 (2)	0.036 (2)	0.045 (2)	0.0000 (19)	0.0154 (19)	0.0032 (18)
N1	0.050 (2)	0.036 (2)	0.107 (3)	0.0023 (18)	0.0183 (18)	−0.0086 (18)
C13	0.052 (3)	0.039 (3)	0.062 (2)	0.003 (2)	0.0088 (19)	0.003 (2)
C11	0.047 (2)	0.044 (2)	0.065 (2)	0.001 (2)	0.013 (2)	0.002 (2)
C14	0.050 (2)	0.036 (2)	0.047 (2)	0.000 (2)	0.0138 (19)	0.0088 (18)
O1	0.0447 (18)	0.159 (3)	0.104 (3)	−0.010 (2)	0.0170 (17)	−0.039 (2)
C21	0.055 (3)	0.053 (3)	0.058 (2)	0.001 (2)	0.0142 (19)	−0.006 (2)
C15	0.052 (2)	0.039 (2)	0.045 (2)	0.000 (2)	0.018 (2)	0.0040 (19)
C19	0.053 (3)	0.057 (3)	0.063 (3)	0.008 (2)	0.010 (2)	0.005 (2)
C20	0.065 (3)	0.052 (3)	0.068 (3)	0.007 (2)	0.022 (2)	−0.011 (2)
C17	0.063 (3)	0.060 (3)	0.059 (2)	0.005 (2)	0.015 (2)	−0.011 (2)
C12	0.051 (3)	0.064 (3)	0.079 (3)	0.003 (2)	0.020 (2)	−0.014 (2)
C18	0.056 (3)	0.080 (3)	0.064 (3)	0.008 (2)	0.002 (2)	−0.013 (2)
C10	0.060 (3)	0.066 (3)	0.075 (3)	−0.004 (2)	0.017 (2)	−0.010 (3)
C7	0.059 (3)	0.082 (3)	0.074 (3)	0.001 (3)	0.017 (2)	−0.003 (3)
C8	0.053 (3)	0.084 (3)	0.084 (3)	0.012 (3)	0.031 (3)	−0.001 (3)
C9	0.089 (4)	0.073 (3)	0.088 (3)	0.003 (3)	0.039 (3)	−0.017 (3)
C1	0.050 (3)	0.104 (4)	0.088 (4)	0.017 (3)	0.000 (3)	−0.024 (3)
C22	0.077 (3)	0.097 (4)	0.129 (4)	0.028 (3)	0.031 (3)	−0.010 (4)
C2	0.073 (3)	0.121 (5)	0.106 (4)	0.014 (4)	0.005 (3)	−0.023 (4)
C5	0.118 (5)	0.142 (6)	0.099 (5)	0.046 (5)	0.015 (4)	0.027 (5)
C4	0.128 (6)	0.151 (7)	0.091 (5)	0.056 (5)	−0.015 (5)	−0.018 (5)
C6	0.082 (4)	0.117 (5)	0.101 (4)	0.013 (3)	0.015 (3)	0.001 (4)
C3	0.100 (5)	0.158 (7)	0.125 (6)	0.022 (5)	−0.006 (5)	−0.039 (6)

### N-[2-(4-Fluoro-3-phenoxybenzoyl)hydrazinecarbothioyl]-4-methoxybenzamide (A2) Geometric parameters (Å, º)

**Table:**

S1—C14	1.661 (4)	C20—H20	0.9300
O3—C15	1.223 (4)	C17—C18	1.377 (5)
O2—C13	1.229 (4)	C17—H17	0.9300
N3—C14	1.383 (4)	C12—C7	1.380 (5)
N3—C15	1.391 (4)	C12—H12	0.9300
N3—H3N	0.8600	C18—H18	0.9300
O4—C19	1.367 (4)	C10—C9	1.392 (6)
O4—C22	1.419 (5)	C10—H10	0.9300
N2—C14	1.322 (4)	C7—C8	1.362 (6)
N2—N1	1.369 (4)	C8—C9	1.360 (6)
N2—H2N	0.8600	C9—H9	0.9300
F1—C8	1.348 (4)	C1—C2	1.365 (7)
C16—C21	1.377 (5)	C1—C6	1.365 (7)
C16—C17	1.390 (5)	C22—H22A	0.9600
C16—C15	1.476 (5)	C22—H22B	0.9600
N1—C13	1.325 (5)	C22—H22C	0.9600
N1—H1	0.8600	C2—C3	1.338 (8)
C13—C11	1.490 (5)	C2—H2	0.9300
C11—C10	1.373 (5)	C5—C4	1.390 (9)
C11—C12	1.383 (5)	C5—C6	1.401 (8)
O1—C7	1.375 (5)	C5—H5	0.9300
O1—C1	1.383 (5)	C4—C3	1.359 (9)
C21—C20	1.374 (5)	C4—H4	0.9300
C21—H21	0.9300	C6—H6	0.9300
C19—C18	1.374 (5)	C3—H3	0.9300
C19—C20	1.378 (5)		
			
C14—N3—C15	128.0 (3)	C11—C12—H12	119.8
C14—N3—H3N	116.0	C19—C18—C17	120.5 (4)
C15—N3—H3N	116.0	C19—C18—H18	119.8
C19—O4—C22	117.7 (3)	C17—C18—H18	119.8
C14—N2—N1	119.4 (3)	C11—C10—C9	119.4 (4)
C14—N2—H2N	120.3	C11—C10—H10	120.3
N1—N2—H2N	120.3	C9—C10—H10	120.3
C21—C16—C17	117.8 (3)	C8—C7—O1	116.6 (4)
C21—C16—C15	118.1 (3)	C8—C7—C12	118.5 (4)
C17—C16—C15	124.1 (3)	O1—C7—C12	124.9 (4)
C13—N1—N2	120.9 (3)	F1—C8—C9	118.9 (4)
C13—N1—H1	119.6	F1—C8—C7	118.8 (4)
N2—N1—H1	119.6	C9—C8—C7	122.3 (4)
O2—C13—N1	121.7 (3)	C8—C9—C10	119.3 (4)
O2—C13—C11	122.9 (4)	C8—C9—H9	120.4
N1—C13—C11	115.4 (4)	C10—C9—H9	120.4
C10—C11—C12	120.0 (4)	C2—C1—C6	121.5 (5)
C10—C11—C13	122.1 (4)	C2—C1—O1	115.1 (5)
C12—C11—C13	117.9 (4)	C6—C1—O1	123.4 (5)
N2—C14—N3	115.4 (3)	O4—C22—H22A	109.5
N2—C14—S1	123.2 (3)	O4—C22—H22B	109.5
N3—C14—S1	121.4 (3)	H22A—C22—H22B	109.5
C7—O1—C1	121.6 (4)	O4—C22—H22C	109.5
C20—C21—C16	121.7 (4)	H22A—C22—H22C	109.5
C20—C21—H21	119.2	H22B—C22—H22C	109.5
C16—C21—H21	119.2	C3—C2—C1	120.4 (7)
O3—C15—N3	120.7 (3)	C3—C2—H2	119.8
O3—C15—C16	122.3 (3)	C1—C2—H2	119.8
N3—C15—C16	116.9 (3)	C4—C5—C6	119.0 (7)
O4—C19—C18	115.0 (4)	C4—C5—H5	120.5
O4—C19—C20	125.6 (4)	C6—C5—H5	120.5
C18—C19—C20	119.3 (4)	C3—C4—C5	120.3 (7)
C21—C20—C19	119.9 (4)	C3—C4—H4	119.9
C21—C20—H20	120.0	C5—C4—H4	119.9
C19—C20—H20	120.0	C1—C6—C5	118.2 (6)
C18—C17—C16	120.8 (4)	C1—C6—H6	120.9
C18—C17—H17	119.6	C5—C6—H6	120.9
C16—C17—H17	119.6	C2—C3—C4	120.6 (7)
C7—C12—C11	120.5 (4)	C2—C3—H3	119.7
C7—C12—H12	119.8	C4—C3—H3	119.7
			
C14—N2—N1—C13	−143.5 (4)	C13—C11—C12—C7	−179.2 (4)
N2—N1—C13—O2	−6.2 (6)	O4—C19—C18—C17	180.0 (4)
N2—N1—C13—C11	173.9 (3)	C20—C19—C18—C17	−0.2 (6)
O2—C13—C11—C10	147.9 (4)	C16—C17—C18—C19	0.7 (6)
N1—C13—C11—C10	−32.2 (5)	C12—C11—C10—C9	−0.6 (6)
O2—C13—C11—C12	−30.6 (5)	C13—C11—C10—C9	−179.1 (4)
N1—C13—C11—C12	149.3 (4)	C1—O1—C7—C8	149.6 (5)
N1—N2—C14—N3	176.7 (3)	C1—O1—C7—C12	−33.4 (7)
N1—N2—C14—S1	−4.1 (5)	C11—C12—C7—C8	−1.8 (6)
C15—N3—C14—N2	−5.7 (5)	C11—C12—C7—O1	−178.8 (4)
C15—N3—C14—S1	175.2 (3)	O1—C7—C8—F1	−2.0 (6)
C17—C16—C21—C20	−0.7 (5)	C12—C7—C8—F1	−179.3 (4)
C15—C16—C21—C20	−179.7 (3)	O1—C7—C8—C9	176.8 (4)
C14—N3—C15—O3	5.9 (5)	C12—C7—C8—C9	−0.4 (7)
C14—N3—C15—C16	−174.1 (3)	F1—C8—C9—C10	−179.1 (4)
C21—C16—C15—O3	5.2 (5)	C7—C8—C9—C10	2.1 (7)
C17—C16—C15—O3	−173.8 (3)	C11—C10—C9—C8	−1.5 (7)
C21—C16—C15—N3	−174.7 (3)	C7—O1—C1—C2	148.1 (4)
C17—C16—C15—N3	6.3 (5)	C7—O1—C1—C6	−34.9 (7)
C22—O4—C19—C18	−177.2 (4)	C6—C1—C2—C3	−2.0 (8)
C22—O4—C19—C20	3.0 (6)	O1—C1—C2—C3	175.1 (5)
C16—C21—C20—C19	1.2 (6)	C6—C5—C4—C3	0.1 (9)
O4—C19—C20—C21	179.1 (4)	C2—C1—C6—C5	1.7 (8)
C18—C19—C20—C21	−0.7 (6)	O1—C1—C6—C5	−175.1 (4)
C21—C16—C17—C18	−0.2 (6)	C4—C5—C6—C1	−0.7 (8)
C15—C16—C17—C18	178.8 (3)	C1—C2—C3—C4	1.3 (9)
C10—C11—C12—C7	2.3 (6)	C5—C4—C3—C2	−0.4 (10)

### N-[2-(4-Fluoro-3-phenoxybenzoyl)hydrazinecarbothioyl]-4-methoxybenzamide (A2) Hydrogen-bond geometry (Å, º)

**Table:**

*D*—H···*A*	*D*—H	H···*A*	*D*···*A*	*D*—H···*A*
N2—H2*N*···O3	0.86	1.92	2.589 (4)	134
N3—H3*N*···S1^i^	0.86	2.80	3.615 (3)	159
C17—H17···S1^i^	0.93	2.69	3.614 (4)	174
N1—H1···O2^ii^	0.86	2.15	2.915 (4)	148
C21—H21···O3^iii^	0.93	2.57	3.399 (4)	148

Symmetry codes: (i) −*x*+1, *y*, −*z*+1/2; (ii) *x*, *y*+1, *z*; (iii) −*x*+1, −*y*−1, −*z*.
